# APPLYING A SALUTOGENIC MODEL TO EXPLORE RESOURCES UTILIZATION IN OLDER ADULTS WITH PREDIABETES: A QUALITATIVE STUDY

**DOI:** 10.1093/geroni/igae098.3404

**Published:** 2024-12-31

**Authors:** Yaqian Liu, Angela Leung, Jed Montayre

**Affiliations:** The Hong Kong Polytechnic University, Hong Kong, Hong Kong; The Hong Kong Polytechnic University, Hong Kong, Hong Kong; The Hong Kong Polytechnic University, Hong Kong, Hong Kong

## Abstract

**Background:**

Older adults with pre-diabetes face unique health challenges, including limited awareness and resources for disease management. The Salutogenic model of Health focuses on identifying and utilising available resistance resources surrounding people to cope with tension created by stressors. This approach is crucial for this vulnerable population addressing both prevention and effective management of pre-diabetes.

**Objectives:**

To explore pre-diabetes population’s experience towards receiving diabetes risk information, and what available resources employed to cope with stressful pre-diabetes situation.

**Methods:**

This descriptive phenomenological qualitative study using six-step inductive thematic analysis was conducted in Hong Kong. Individual in-depth semi-structured interview guided by Salutogenic model were conducted to explore the experience of coping with prediabetes through the lens of sense of coherence (comprehensibility, manageability, meaningfulness), available resources and their utilization. Purposive sampling was used to select adults above 65 years diagnosed with pre-diabetes.

**Results:**

The study revealed that older adults with pre-diabetes primarily leverage personal resilience, community support, and healthcare guidance as coping resources. Comprehensibility was enhanced through tailored health education, enabling participants to grasp their condition’s implications. Manageability was facilitated by accessible healthcare services and social support networks, aiding in lifestyle adjustments. Meaningfulness emerged as participants perceived their proactive health behaviors as future investments. Despite varying levels of resource accessibility, a common thread was the pursuit of a balanced, informed approach to pre-diabetes management.

**Conclusion:**

This study provided resource map and coping mechanisms of pre-diabetic population in Hong Kong. This informs the development of a Resources-Oriented Salutogenic intervention tailored to prediabetes population.

